# Establishing and Sustaining a Prospective Screening Program for Breast Cancer-Related Lymphedema at the Massachusetts General Hospital: Lessons Learned

**DOI:** 10.3390/jpm5020153

**Published:** 2015-05-20

**Authors:** Cheryl Brunelle, Melissa Skolny, Chantal Ferguson, Meyha Swaroop, Jean O’Toole, Alphonse G. Taghian

**Affiliations:** 1 Department of Physical and Occupational Therapy, Massachusetts General Hospital, Boston, MA 02114, USA; E-Mails: cbrunelle@partners.org (C.B.); jotoole244@comcast.net (J.O.); 2 Department of Radiation Oncology, Massachusetts General Hospital, Harvard Medical School, Boston, MA 02114, USA; E-Mails: mskolny@partners.org (M.S.); cferguson4@partners.org (C.F.); mswaroop@partners.org (M.S.)

**Keywords:** breast cancer-related lymphedema, lymphedema, screening, perometer, breast cancer

## Abstract

There has been an increasing call to prospectively screen patients with breast cancer for the development of breast cancer-related lymphedema (BCRL) following their breast cancer treatment. While the components of a prospective screening program have been published, some centers struggle with how to initiate, establish, and sustain a screening program of their own. The intent of this manuscript is to share our experience and struggles in establishing a prospective surveillance program within the infrastructure of our institution. It is our hope that by sharing our history other centers can learn from our mistakes and successes to better design their own prospective screening program to best serve their patient population.

## 1. Introduction

As the long-term survival rate for breast cancer patients improves, there is an increasing focus on helping survivors manage the sequelae of breast cancer treatment that may affect function and/or quality of life. Currently there are more than 2.8 million breast cancer survivors in the United States, and it is estimated that approximately one in five will develop breast cancer-related lymphedema (BCRL) [1,2]. The risk of developing BCRL remains for a lifetime and it is one of the most feared side effects among breast cancer survivors. There is currently no cure and patients who develop BCRL are often subjected to a long-term struggle of trying to manage their edematous limb with bandaging, compression garments, and self-care through treatment by a Certified Lymphedema Therapist. Previous analyses of our patient population has suggested that individuals who undergo axillary lymph node dissection (ALND), regional lymph node radiation (RLNR) or have a high BMI at the time of their breast cancer diagnosis are more likely to develop BCRL [3,4,5]. This has helped us to educate patients and to understand which patients may benefit most from close surveillance.

The 2012 National Lymphedema Network position paper “Screening and Early Detection of Breast Cancer-Related Lymphedema: The Imperative” [6] addresses the rationale for lymphedema screening as a method to detect and subsequently treat lymphedema at an early, even subclinical stage, so as to “reverse the progression to chronic, irreversible lymphedema.” If early identification of increased fluid in the arm (using a measuring tape for arm circumference, perometry or bioelectric impedance spectroscopy) allows a patient to be identified and managed in a manner that minimizes lymphedema progression, then screening for lymphedema will be advantageous. In 2011, Stout *et al* reported on the direct costs involved with a surveillance approach, compared to the traditional approach of waiting to treat lymphedema until after it has developed [7]. Using a 2009 Medicare physicians’ fee schedule, the projected cost per year per patient of managing lymphedema in a traditional manner was approximately five times greater than in a prospective screening model ($3124.92 *vs.* $636.19 USD). Thus, a prospective surveillance approach would not only have a positive impact on the patient, but also reduce financial burden on the healthcare system.

In 2014, Ostby *et al.* proposed a detailed framework for a prospective surveillance program for BCRL (PROSURV-BCRL), and rightly stated that such a model is “necessary in order to detect BCRL at an early stage when there is the best chance to decrease risk or slow progression” [8]. Despite consensus amongst those treating BCRL that a prospective surveillance model is necessary, beginning and sustaining such a program presents some challenges.

It has been our experience at Massachusetts General Hospital that a prospective surveillance model for BCRL is both feasible and sustainable. Such a screening program, in which patients are continuously monitored for arm volume changes via perometer measurements preoperatively and every few months postoperatively, was implemented at our institution several years ago. We have since screened 4203 patients at pre-operative baseline and 2598 patients with at least three post-operative measurements.

The purpose of this paper is to describe and share our experience creating, implementing, and sustaining a prospective lymphedema screening program at a large academic medical center. While we understand that each institution that treats lymphedema is a unique entity and what is feasible in our institution may not be possible in another’s, we hope that by sharing our struggles and success we can help other institutions establish lymphedema screening programs that ultimately contribute to improving lymphedema detection and the quality of life of breast cancer survivors.

## 2. History of the Breast Cancer-Related Lymphedema Screening Program at Massachusetts General Hospital

The lymphedema studies program was initiated in 2005, with the dual purpose of providing clinical care via prospective screening to the breast cancer population, as well as pursuing research-related questions pertaining to risk factors, incidence, and treatment for this chronic condition. Initially founded by a radiation oncologist (AGT) and a certified lymphedema therapist (JAO), the team has evolved to include a multidisciplinary team of breast medical and surgical oncologists, certified lymphedema therapists, nurse practitioners, and patient advocates. There was a mutual commitment by members of the oncology and physical therapy departments to create and implement a multidisciplinary team to address and treat this unfortunate side effect of breast cancer treatment. Administrative leaders from both departments were very dedicated to establishing this collaboration which helped make establishing such a program possible. At the time of the program’s initiation, financial support was provided by philanthropic donations. This covered the cost of purchasing a perometer, adjustable exam table, and the salary of a research assistant to perform the perometer measurements and track patients. In 2008, the program was awarded two federal grants for two clinical trials; this supported the program for a period of five years. During this time, we opened two additional clinical trials and our research staff grew to include two research assistants and one program manager. Currently, the program is supported by the Adele McKinnon fund for Breast Cancer-Related Lymphedema which supports the salaries of these individuals. The salaries of the professional staff members and licensed medical personnel are covered by their respective departments. These individuals are dedicated to the program’s cause and volunteer their time to the program.

### Mode of Limb Volume Measurement: The Perometer

**Figure 1:**
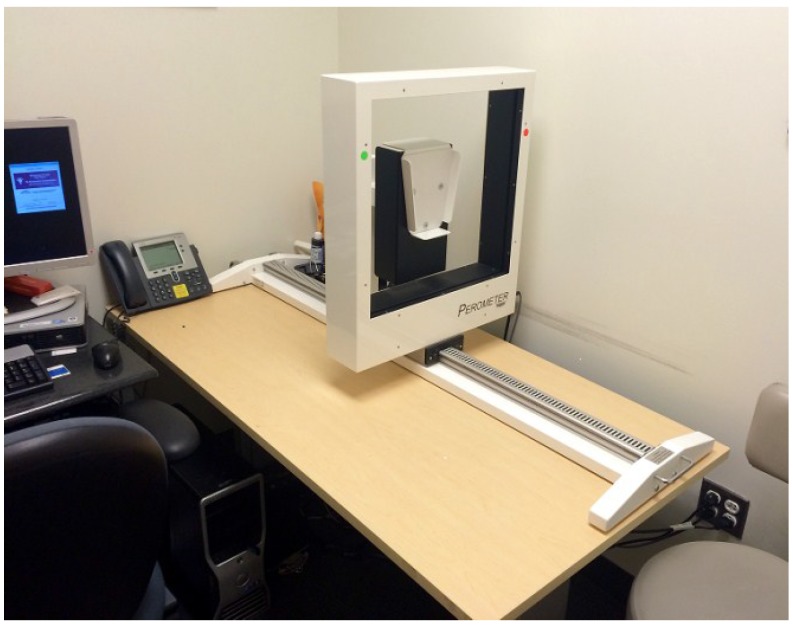
Massachusetts General Hospital (MGH) perometer room.

After researching the various measurement options utilized in the literature, a horizontal perometer was purchased in 2005. A perometer was chosen based on its quick and accurate ability to measure patients’ limb volume. This would allow us to screen large volumes of patients without disrupting the current clinic workflow or extending the time of the patient’s oncology appointment. Obtaining a designated clinical space within the breast center for the perometer was challenging. Similar to other health centers, clinical space is very limited at our institution. Given the commitment of the team, they were able to secure a small space (7.5 × 5 feet or 2.3 × 1.5 m) within the Breast Center that could house the perometer (Figure 1).

This centralized location was ideal as it was within close proximity to the breast cancer patients who were seeing their oncology providers in the clinic. Additionally, an adjustable table was purchased that would accommodate different patient heights to maintain a standard protocol for measurement. Given the large volume of patients seen at MGH, it was decided that a designated individual would be hired to obtain the perometer measurements and track patients. If available resources do not include a dedicated individual for perometry, measurements could be done by anyone who is trained, is detail-oriented, and is working in close proximity to the perometer.

While the most accurate mode of measurement is still debated amongst researchers and clinicians, it is important to remember that any standardized prospective assessment is better than none at all. Different measures of arm volume, circumference, or edema (perometry, water displacement, circumference with a tape measure with or without arm volume calculated with computer software, bioelectrical impedance spectroscopy) have been shown to be valid; however, they are not interchangeable and efforts should be made to have the same practitioner complete measurements across time in the same patient [9,10,11,12,13,14]. Efforts can be made to improve the accuracy of each measurement method by standardizing measurement protocols.

## 3. Establishing a Screening Protocol

When the team began measuring patients with perometry to assess for BCRL in the fall of 2005, very little information was available regarding screening patients for BCRL. Patients were not measured per a standardized time schedule, but would rather undergo periodic perometer measurements at the discretion of the treating oncologists. We did not know the importance of the pre-operative baseline measurement and patients would undergo their first measurement pre or post operatively. We also did not realize that consistent periodic measurements would provide important information that would allow providers to assess trends related to increases in the patient’s arm volume that may be an early sign of BCRL development.

### 3.1. Establishing a Timeline for Limb Volume Measurement

An initial review of the bilateral pre-surgical baseline perometer measurements indicated that approximately 20% of patients had a natural asymmetry between their arms, including 11.2% for whom the baseline volume of the ipsilateral arm exceeded that of the contralateral arm by at least 5% [15,16]. This asymmetry was unrelated to hand dominance. Without knowledge of this asymmetry it would be difficult to determine the true volume difference between a patient’s limbs. Therefore, it was determined that all newly-diagnosed patients would initiate their lymphedema screening at the time of their pre-operative consultation with their breast oncology multidisciplinary team. The preoperative measurement would serve as the patient’s baseline to use as a comparison for all future measurements. A subsequent measurement should occur at the patient’s post-operative appointment to quantify and document any post-operative swelling. A previous study by Specht *et al* [17] determined that post-operative swelling of ≥3% within three months predicted potential future BCRL development. The data collected from these two measurements will be crucial when assessing for future changes in the patient’s limb volume and for determining a patient’s individual risk for developing BCRL. Regardless of the measurement schedule, it is imperative that all patients undergo at least a pre- and post-operative assessment. Our clinical experience and research has suggested that longitudinal measurement series are best for determining natural history patterns of arm volume increases that may be indicative of BCRL development [17]. Screening initiation occurs at the time of the patient’s preoperative visit with the patient being routinely assessed while they are actively undergoing breast cancer therapy. Following completion of their breast cancer treatment, the patient will be assessed approximately every six months (range 3–7 months) in conjunction with their follow-up appointments with their breast oncologists. This time frame was determined based on the frequency and time interval between appointments that occurred at our institution. This would allow us to routinely screen patients while they were already at the hospital for oncology follow-up visits. The majority of patients treated at MGH have both their surgery and any adjuvant therapies at our institution. A small number of patients receive only their adjuvant therapy at MGH. These patients are screened at the discretion of their treating oncologist. BCRL screening continues throughout the duration of the patient’s treatment and follow-up care, regardless of the patient’s individual risk for developing BCRL. We currently have 2656 patients with at least three measurements and the median follow-up time of 28.53 months post-operatively. Previous analysis of our patient cohorts indicates that the median time to BCRL development is 18–24 months post-operatively. Regardless of a patient’s natural arm volume, trends or associated risk factors, all patients are routinely followed.

### 3.2. Establishing a Limb Volume Measurement Protocol

Initially, the perometer measurements were not performed in a standardized fashion with regard to how the hand and arm were positioned. After reviewing the measurement series captured on these early patients, it was determined that in order to accurately measure arm volume and therefore diagnose BCRL, there needed to be a standardized measurement protocol for all patients with breast cancer. After evaluating various approaches to performing the perometer measurements with regard to arm and hand position, a standardized protocol was established. This measurement protocol was previously published [16]. In brief, patients are positioned sitting in a chair perpendicular to and at the end of the perometer table with the shoulder abducted 90 degrees, elbow in full extension, and wrist at neutral, with palm down and fingers and thumb together and straight, so the hand is flat. Table height is adjusted so that the arm is parallel to the exam table holding the perometer. In this position, the perometer measurement is taken from the finger tips to the axillary crease. The frame is moved at a slow and steady rate across the extremity. The length of the measurement, which corresponds to the length of the patient’s arm, is adjusted per patient and standardized for each measurement. This creates consistency in the measurements, as the patient’s limb is measured at the same length for all measurements on the perometer. Each patient undergoes serial bilateral arm measurements. Three measurements are performed on each limb. The median arm volume per each limb is included in the arm volume change quantification formulas. For unilaterally-affected patients, this is the relative volume change (RVC) equation. For bilaterally-affected patients, it is the weight-adjusted change (WAC) equation [15,16,18,19,20].

### 3.3. Calculating Limb Volume Change over Time

In 2008, the MGH lymphedema studies team developed a formula that would determine a patient’s arm volume change while incorporating temporal changes and any pre-existing asymmetry [16]. The relative volume change equation was validated and published in 2010. It calculates the relative change in arm volume of the affected limb to the pre-operative assessment and accounts for change in size of the contralateral arm as a control. Because the contralateral arm serves as the control, this formula can only be used in patients who underwent unilateral surgery. In 2013, our group validated and published the weight-adjusted change formula to be utilized in patients who underwent bilateral breast surgery. Similar to the RVC formula, this equation functions independently of the contralateral arm and accounts for fluctuations in patients weight. Briefly, RVC = [(A(2)U(1)/U(2)A(1)) − 1] where A(1) and A(2) are arm volumes on the surgical side at pre- and post-operative measurements and U(1) and U(2) are arm volumes on the contra-lateral side at corresponding time points [15]. The WAC calculates changes in arm volume compared to a pre-operative arm measurement for each arm independently and accounts for weight changes that could influence arm size [19]. At our institution, the arm volume obtained by the perometer is included in each of the respected formulas. This value, as well as the patient’s raw arm volumes, are entered into our hospital’s electronic medical record for the provider.

### 3.4. Integration of Perometry Screening into the Clinic Workflow

Early in the program, there was some concern that perometer measurements could interrupt the workflow and clinic schedule. With time, perometry became an integral part of the care of these patients (alongside vital signs) and is woven into the garment of the clinic so as not to interrupt workflow. Patients who are due for their periodic perometer measurement are identified and informed that they need a perometer measurement when they arrive at the clinic. After their visit with the provider, the medical or research assistant escorts them to the perometer room for a measurement.

For newly-diagnosed patients, screening initiation occurs at the time of the patient’s first pre-operative consult with the oncology team. Patients are provided with an information packet on BCRL screening as they check in for their appointment. This information is accompanied by other material related to their breast cancer treatment and other supportive programs offered by the hospital. This packet provides a brief explanation of BCRL and how and why we screen for this side effect of treatment. Prior to seeing the oncology team, a member of the lymphedema studies team meets with the patient to perform the perometer measurement. The team member further explains why we screen for BCRL and how the perometer works. The team member also explains what signs and symptoms they will be evaluating for when assessing for the presence of BCRL. Lastly, the team member informs the patient of the measurement schedule and when their next measurement will occur. The purpose at the pre-operative visit is to introduce the concept of BCRL briefly and for the patient to understand how and why we are measuring her arms with perometry and how we will be screening for lymphedema throughout her time at MGH. Since patients are measured before they see their oncology team, treatment plans have not been discussed with the patient and therefore potential risk factors are unknown at this point. Therefore, we do not discuss the patient’s risk of developing lymphedema at the pre-operative visit. At the time of the patients’ post-operative visit with their oncology team, recommendations or risk-reduction practices are discussed with the patient. At the time of the patient’s post-operative visit, which can occur between two and six weeks after surgery, the surgical oncology nurse practitioners review range-of-motion exercises with the patients to help restore full range of motion. At this time, BCRL education, including risk factors, early signs and symptoms of the condition, and risk-reduction activities are reiterated with the patient. The patient is advised to contact the treating oncologist immediately if there are signs or symptoms of BCRL, and contact information is given.

The multidisciplinary oncology team at MGH collects and documents detailed clinical information at follow-up appointments: history and physical exam, including height, weight, body mass index, limb volume measures via perometry, skin assessment, general physical exam, and comment on function and current condition. At MGH, each patient is educated and referred as appropriate to resources available within MGH or the community; for example social work, nutrition, physical therapy, occupational therapy, or support groups, alternative therapies, or workshops. Ostby *et al.*’s PROSURV-BCRL recommendations [8] outline a detailed summary of data collection and interventions in such a model.

### 3.5. Transient Edema

After years of screening patients, it is our clinical experience that some patients will experience a periodic increase in arm volume that resolves without treatment intervention. A previous study by Kilbreath *et al.* has suggested that transient edemas in the breast cancer population do occur and self-resolve without the need for therapy [21]. This is an important point to consider when assessing a patient’s risk for developing BCRL. A patient’s own individual risk factors and previous trends in each patient’s arm volume measurements should be considered when assessing for a patient’s risk for progression into a larger more chronic BCRL. Patients who demonstrate a low-level volume increase, defined as a 5%–10% change from baseline, who possess at least one of the known BCRL risk factors (ALND, RLNR and high BMI) [3,4,5] are asked to return to clinic within 4–7 weeks for a reassessment. Other than education, these patients receive no interventions for edema during that time. A majority of patients at this subsequent measurement are found to have a decrease in arm volume. However, patients whose arm volume remains elevated and/or are having symptoms of BCRL are referred to their oncologists for further assessment and consideration of a Certified Lymphedema Therapist (CLT) consult.

### 3.6. Implementation of a Lymphedema Screening Trial

Data from the first few years of the screening protocol allowed us to assess the natural history of BCRL, evaluate rates of progression, and determine the relative volume change to test for appropriate intervention. With this information, we were able to design and implement a prospective clinical BCRL screening trial that simultaneously assesses quantifiable arm volume changes and self-reported symptoms and changes in QOL (ClinicalTrials.gov Identifier: NCT01521741). Patients who enroll into this trial are asked to complete a comprehensive questionnaire that assesses the multiple QOL (physical, emotional, and functional) domains that are impacted by BCRL. This study has provided valuable insight into the impact of breast cancer treatment on arm functionality and ability to perform activities of daily living. Additionally, it has allowed the team to assess the relationship between cording and BCRL development, as well as the impact of fear-avoidance behavior on QOL [22,23,24].

## 4. MGH Screening Program—Certified Lymphedema Therapist

The CLT’s at MGH include physical therapists and an occupational therapist. The treatment for patients with BCRL is therefore billed as physical or occupational therapy. The nurse practitioners and physicians in the Breast Center are the most frequent providers referring to the CLT’s at MGH. Other referrals come from Plastic Surgery, Medical and Radiation Oncology, mostly via nurse practitioners in those departments. The two most common reasons for referral to the CLT include BCRL and impaired shoulder mobility after surgery or radiation. Some patients are referred for subclinical lymphedema (RVC or WAC < 10%), especially in situations when the patient has had repeated measures of an RVC or WAC 5%–10%, associated risk factors for BCRL and/or is in need of further education about BCRL. In general, patients are immediately referred to the CLT if perometry is ≥10% RVC or WAC on any given measure. The goal is to evaluate the patient within one to two weeks, and frequently the patient is seen sooner.

The team approach is central to the care of patients with breast cancer at MGH [25]. The team members communicate closely and respond to one another in a very timely fashion, whether that is the CLT concerned that a patient may have developed cellulitis, or a nurse practitioner wondering if there is a CLT available nearer a patient’s home, for example. This team approach allows the team to treat the patient without delays in care, it allows for consistent care in the breast center by providers who know the patient (e.g., instead of sending a patient to the Emergency Department with suspected cellulitis, the nurse practitioner may immediately examine her), and collaboration in establishing the best plan of care for each patient.

Patients often comment to the CLT that BCRL is an integral part of conversations with the disciplines. Patients understand the importance of surveillance and frequently seek out perometer measurements if any symptoms develop. These readings are available to them at any point and they can simply call for or come in for an appointment. These measurements are part of the patient’s treatment and are not billed; they are covered by research grants and philanthropy. Arm volume is shared with the patient at the time of measurement, and the CLT is paged with the measurement if the patient is on service, or a referral is mobilized if necessary.

The decision to locate the CLT within the physical therapy department was a conscious one. Primarily, there is limited space in the Breast Center, and there are more resources for treatment in the PT and OT department, including private treatment space, as well as a space for compression supplies. Secondly, there is a team of CLT’s built within the other therapists, and these therapists treat patients other than those with BCRL, so location within the department is a necessity. Finally, location within the PT and OT department nourishes the relationship between the breast center and the physical therapy department. The team CLT has many opportunities to enhance the relationship with the Breast Center, including attendance at Breast Rounds, Tumor Board, and at NP meetings. Comprehensive and concise documentation by all in the electronic medical record is integral to successful communication within the multidisciplinary team treating these patients. The location of the CLTs in the PT and OT department, rather than in the Breast Center, has not been a barrier to the program.

When treatment for BCRL is not geographically feasible at MGH, the CLTs at MGH help the patient locate a CLT closest to home. Feasibility of getting to treatment and developing a long-term rapport with a CLT is important for patients diagnosed with BCRL given its chronicity and need for recurrent treatment.

## 5. Funding for Compression Garments

Given that Medicare and Medicaid do not provide coverage for compression garments for patients with BCRL, financial support is necessary for patients who are unable to afford them. These garments are expensive and replaced at least yearly for patients who wear them frequently, and this becomes an expensive, yet uncovered, medical necessity. Becoming part of the community and connecting with clubs or programs that have adopted breast cancer or BCRL as a charitable mission has made it possible to fund grants for compression garments over the years. This money ensures that we can provide compression garments to any patient who needs them. The team continues to work on sustainability of this fund with the various hospital resources. Funding for garments and sustainability of the fund would be an important area of focus for any prospective surveillance model.

## 6. Lessons Learned

Through implementation of a prospective surveillance model for BCRL, we have had the opportunity to learn through our mistakes and through our data. We now understand that our patients should be screened with a strict measurement protocol, in a longitudinal manner, starting with at least a pre-operative baseline and a post-operative measurement, on a standard schedule, which aligns with clinical follow-up appointments. We know that the median time to development of BCRL has been demonstrated to range from 18–24 months, and our follow-up continues for as long as patients are followed in the hospital. We know that calculation of limb volume must take into account fluctuations in body weight and the baseline volume of both upper extremities. We understand which patients are at higher risk of developing BCRL after treatment: this includes patients who have undergone axillary lymph node dissection, regional lymph node radiation, or who have a high BMI (>30) at diagnosis or >3% edema in the post-operative period (*i.e.*, within three months) or 5%–10% edema at any time as well as those who develop cellulitis at any time. These patients will likely benefit from closer surveillance. Therefore, we also understand which patients are at lower risk of developing BCRL after treatment; perhaps, we need to study whether we can monitor these patients less frequently without increased risk of missing an early diagnosis of BCRL. We understand that, as clinicians screening for and treating patients with BCRL, we identify clinical questions and gaps in the literature. We also think that standardization of the definition and measurement of BCRL is desperately required to strengthen the research base. In 2008, the program was awarded two federal grants for a prospective longitudinal screening trial. By obtaining additional funding to cover the costs of the program’s research efforts, the lymphedema screening program evolved to contain an important research component that allowed us to evaluate our large patient data sets that we had accumulated since the initiation of the screening trial. Through various analyses of our data, we have examined the impact of breast cancer treatment on arm function and ability to perform activities of daily living, the relationship between cording and BCRL development, the impact of fear-avoidance behavior on QOL and both treatment and non-treatment associated risk factors of BCRL. We know that in today’s healthcare climate, proving the positive quality of life impact, as well as the cost savings of a prospective screening model, is necessary in order to justify and sustain a place for such a model in the care of breast cancer survivors. It will therefore be important for the community to come together and collaborate to do research in order to generate level I evidence through multi-institutional clinical trials. Finally, we understand that BCRL is one of many sequelae of breast cancer with which these patients need to cope, and not the only outcome deserving of our attention.

## 7. Summary

Current literature strongly advocates for prospective surveillance model approach to BCRL. As clinicians agree that prospective screening is the best approach to this chronic condition, the question still remains of how to best go about successfully implementing such a program. It has been our experience at MGH that such a program is feasible and sustainable. We hope that, by sharing our experience of our struggles and success, we can help other institutions establish BCRL screening programs that ultimately contribute to improving BCRL detection and the quality of life of breast cancer survivors.
